# One-Pot Synthesis of Co-Based Coordination Polymer Nanowire for Li-Ion Batteries with Great Capacity and Stable Cycling Stability

**DOI:** 10.1007/s40820-017-0177-x

**Published:** 2017-12-08

**Authors:** Peng Wang, Xiaobing Lou, Chao Li, Xiaoshi Hu, Qi Yang, Bingwen Hu

**Affiliations:** State Key Laboratory of Precision Spectroscopy, Shanghai Key Laboratory of Magnetic Resonance, School of Physics and Materials Science, East China Normal University, Shanghai, 200062 People’s Republic of China

**Keywords:** Nanowire, Coordination polymer, Lithium-ion battery, Anode, Ultra-high capacity

## Abstract

Nanowire coordination polymer cobalt–terephthalonitrile (Co-BDCN) was successfully synthesized using a simple solvothermal method and applied as anode material for lithium-ion batteries (LIBs). A reversible capacity of 1132 mAh g^−1^ was retained after 100 cycles at a rate of 100 mA g^−1^, which should be one of the best LIBs performances among metal organic frameworks and coordination polymers-based anode materials at such a rate. On the basis of the comprehensive structural and morphology characterizations including fourier transform infrared spectroscopy, ^1^H NMR, ^13^C NMR, and scanning electron microscopy, we demonstrated that the great electrochemical performance of the as-synthesized Co-BDCN coordination polymer can be attributed to the synergistic effect of metal centers and organic ligands, as well as the stability of the nanowire morphology during cycling.

**Electronic supplementary material:**

The online version of this article (10.1007/s40820-017-0177-x) contains supplementary material, which is available to authorized users.

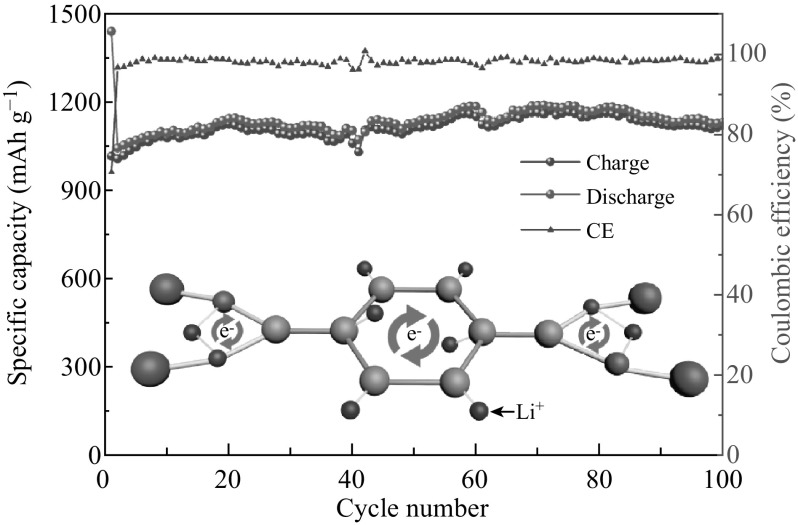

## Highlights

- Amide-group-coordinated cobalt–terephthalonitrile (Co-BDCN) coordination polymers, with a diameter distribution of 45–55 nm, were synthesized by a one-pot solvothermal method.
- Reversible capacity of 1132 mAh g^−1^ was achieved at a current density of 100 mA g^−1^.

## Introduction

Rechargeable lithium-ion batteries (LIBs) have been finding increasing number of applications in a variety of fields, including portable electronic devices, electrical energy storage (EES), electric vehicles (EVs), and hybrid electric vehicles (HEVs) [1]. Given the commercial anode graphite possesses a low theoretical capacity of 372 mAh g^−1^, developing high-capacity anode materials is vital for aforementioned applications, particularly in areas where application of miniaturization is increasing. To develop futuristic high-performance anode materials, stable structure with abundant lithiation sites is necessary. To this end, metallic oxide-based [2–10], Sn-based [11, 12], Si-based [13, 14], and P-based [15–18] anode materials have been widely studied. Although their theoretical capacity is high, their cycling stability is poor due to large volume changes during the charge–discharge process [19–22].

Metal organic frameworks (MOFs) or coordination polymers (CPs), which are assembled by inorganic metal ions as vertices and organic ligands as linkers, have attracted tremendous attention in recent years [23–25]. By varying the metal centers and functional linkers, MOFs with various pore sizes and structures can be designed to cater for the increasing demands in the fields of catalysis, sensing, gas storage, drug delivery, and proton conductivity [26, 27]. Recently, the electrochemical applications, especially for LIBs, have attracted significant attention due to their tremendous potential as both cathode [28–30] and anode materials [31–35]. MOF-177 [36], Zn_3_(HCOO)_6_ [37], Mn-LCP [38], Mn-BTC [39], Co_2_(OH)_2_BDC [40], BiCPs [41], and CoBTC [42] have been applied as anode (Table S1). For example, Co_2_(OH)_2_BDC exhibited reversible capacity of 650 mAh g^−1^ after 100 cycles at current density of 50 mA g^−1^ [40]. In these CPs or MOFs, carboxylate groups (e.g., 1,3,5-benzenetricarboxylate and 1,4-benzenedicarboxylate) are usually used to coordinate with different metal centers (e.g., Mn, Co, and Zn). During the charging process, Li^+^ ions are inserted mainly to the organic moiety (including the carboxylate group and the benzene ring) in these MOFs [39, 42]. The electron-donating effect of the carboxylate group and the benzene ring is considered the main impetus in storing lithium ions. Conjugated dicarboxylates can eventually serve as anode materials without any metal center [43]. However, CP- or MOF-based electrodes with other kinds of organic linkers are seldom used.

Herein, we selected terephthalonitrile as the organic linker and Co(NO_3_)_2_·6H_2_O as the metal source, and synthesized an amide-group-coordinated CP with nanowire-like structure using a simple solvothermal method. The coordination participation of the amide group showed a higher Li^+^ storage performance as compared to Co_2_(OH)_2_BDC, which uses the carboxylate group as a linker. A reversible capacity of 1132 mA g^−1^ was retained after 100 cycles at a rate of 100 mA g^−1^. The synergistic effect between the organic linker and the Co^2+^ center, as well as the excellent stability of the nanowire-like structure, may account for the superior electrochemical performance.

## Experimental

### Materials Synthesis

Co-BDCN was solvothermally synthesized with Co(NO_3_)_2_·6H_2_O (5 mmol, Aladdin, 99.99%) and terephthalonitrile (5 mmol, Aladdin, 99%) in N,N-dimethylformamide (DMF, 50 mL, Sinopharm, AR) solution. The reactants were stirred for 10 min at room temperature to achieve complete dissolution and then transferred to a 100 mL Teflon-lined stainless steel autoclave before heating at 150 °C for 3 or 24 h. The samples obtained after 3 and 24 h will be referred to as Co-BDCN-3h and Co-BDCN-24h, respectively. After cooling to room temperature, the product was filtered and successively washed by DMF and ethanol for three times to remove surplus reactants. The product was finally obtained by drying at 70 °C for 12 h. It is noteworthy that the direct synthesis of Co-BDCN-24h from terephthalamide and Co(NO_3_)_2_·6H_2_O failed due to the very low solubility of terephthalamide in the available solvents (DMF, methanol, alcohol, and water).

### Materials Characterizations

A Rigaku Ultima IV X-ray diffractometer (XRD) with Cu-Kα radiation (*V* = 35 kV, *I* = 25 mA, *λ* = 1.5418 Å) was used to analyze the crystal phase of the as-prepared materials. N_2_-sorption isotherms and BET surface area were measured at 77 K with a 02108-KR-1 system (Quantachrome). The morphologies of the samples were characterized by scanning electron microscopy (SEM, Hitachi S-2400, Japan). Before initiating the test, the samples were mounted on aluminum stubs and sputtered with gold. Thermogravimetric analysis (TGA) was performed using a STA 449 F3 Jupiter^®^, which simultaneously acted as a thermo-analyzer. Temperature was varied from room temperature to 800 °C at a heating rate of 10 °C min^−1^. A Nicolet-Nexus 670 infrared spectrometer was used to perform Fourier transform infrared spectroscopy (FTIR) analysis. The cells for ex situ SEM test were cycled 50 times and discharged to 0.01 V to reduce the reactivity of the electrode. After that, we disassembled the battery in a glove box filled of pure argon and washed the electrode several times with DMC to remove the residual electrolyte. The electrode was tailored and pasted in conductive carbon adhesive tape directly before the test. The inductively coupled plasma (ICP) test was performed on Thermo IRIS Intrepid II XSP spectrometer. Varian 700 M was used to collect ^1^H nuclear magnetic resonance (^1^H-NMR) spectra in liquid state. About 1-mg samples were dispersed in 0.5 mL DMSO-6d. Then the liquids were heated at 80 °C for 5 min and ultrasonically vibrated for 5 more minutes before the ^1^H-NMR test. Bruker 600 M was used to collect ^1^H-NMR spectra in the solid state.

### Battery Performance Measurements

All electrochemical measurements were taken at room temperature. The active material (weight ratio: 80%), conducting additive (Super-P carbon black, weight ratio: 10%), and the binder (carboxymethyl cellulose sodium or CMC, weight ratio: 10%) were homogenously mixed in deionized water (solvent) for at least 3 h to produce a slurry. The thus-obtained slurry was coated onto Cu foil and dried at 70 °C in vacuum oven for 12 h. The electrodes were punched into round plates (diameter of 14.0 mm). The loading of the as-prepared electrodes is about 1.0 mg cm^−2^. 1 M LiPF_6_ in EC–DMC–EMC (1:1:1 in volume) was used as the electrolyte. Finally, a coin cell (CR2032) was assembled by the as-prepared anode, a Celgard 2325 separator (diameter of 19.0 mm), a pure lithium wafer (counter electrode), and electrolyte in an argon-filled glove box, with oxygen and water contents less than 0.1 ppm. The galvanostatic charge and discharge and rate tests were performed on a LAND 2001A battery test system in the voltage range of 0.01–3.0 V. Cyclic voltammetry (CV) and electrochemical impedance spectroscopy (EIS) were performed on an electrochemical workstation (CHI660e) at a scan rate of 0.2 mV s^−1^ in the voltage range of 0.01–3.0 V.

## Results and Discussion

**Fig. 1:**
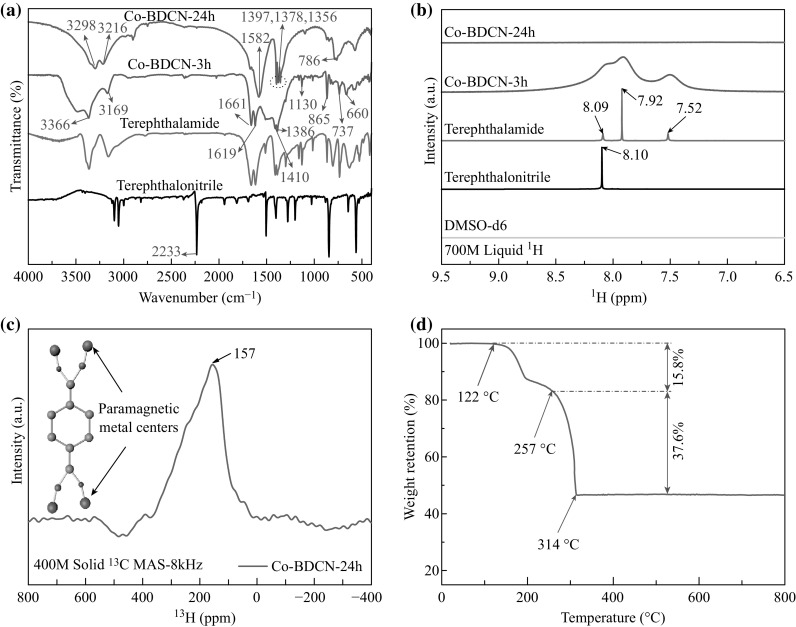
**a** FTIR spectra of Co-BDCN-24h, Co-BDCN-3h, terephthalamide, and terephthalonitrile. **b** ^1^H NMR spectra of Co-BDCN-24h, Co-BDCN-3h, terephthalamide, and terephthalonitrile (dissolved in DMSO-d6 liquids) and DMSO-d6 in liquid state. **c** Solid-state ^13^C NMR spectra of Co-BDCN-24h. **d** TG curves of Co-BDCN-24h

**Scheme 1:**
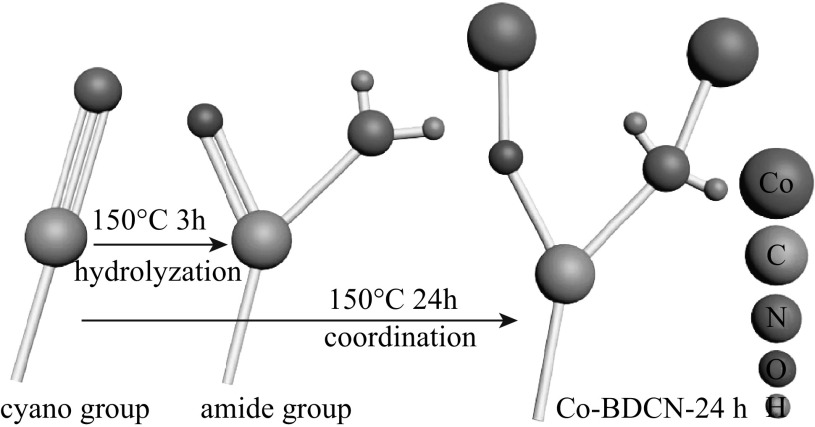
The forming process of Co-BDCN-24h

FTIR is a convenient tool to study the binding patterns of organic linkers and Co^2+^. As shown in Fig. 1a, the sharp absorption band at 2233 cm^−1^ of terephthalonitrile corresponds to *ν*(C≡N); however, no absorption of C≡N can be observed in Co-BDCN-3h and Co-BDCN-24h, indicating the disappearance of C≡N after reaction. For Co-BDCN-3h, the new peaks at 1661, 1619, 1410, and 1386 cm^−1^ can be assigned to the *ν*(C=O) stretching mode (or amide I), amide II, *ν*(C–N), and amide III, respectively, while the absorption band at 865 cm^−1^ can be ascribed to the *ν*(C–C) stretching vibration of Ar–C=O. Besides, two characteristic bands of *ν*(NH_2_) stretching vibration are observed at 3366 and 3169 cm^−1^, whereas the weak absorptions at 1130 and 735–660 cm^−1^ correspond to the *ν*(NH_2_) rocking vibration. The aforementioned absorption bands can be found in pure terephthalamide. However, for Co-BDCN-24h, new peaks appeared at 1582 cm^−1^ and can be assigned to the asymmetric stretching vibration of C=O. The peaks at 3298–3216, 1397, 1378, and 1356 cm^−1^ can be assigned to *ν*(NH_2_) stretching vibration, *ν*(C–N), amide III and symmetric stretching vibration of C=O, respectively. The redshift of *ν*(C=O) and the variations of *ν*(NH_2_) are due to the participation of amide group in coordination. These facts corroborate the hydrolysis of cyano group to amide group in a mass hydrothermal process [44] and subsequent coordination of Co^2+^ with amide in Co-BDCN-24h, as depicted in Scheme 1.

The ^1^H NMR spectra in liquid state are shown in Fig. 1b. The chemical shift of H in -D_2_H of DMSO-d6 was set at 2.5 ppm (Fig. S1). A well-defined peak is detected at 8.10 ppm for the H (–Ar) of terephthalonitrile, while three resonances of intensity ratio of 1:2:1 at 7.52 ppm (H_a_, –NH_2_), 7.92 ppm (H, –Ar), and 8.09 ppm (H_b_, –NH_2_) are observed in the terephthalamide (the two protons in –NH_2_ of the amide group show different chemical shifts due to magnetic anisotropy, electric field, and steric effects) [45]. For the formation of amide groups in Co-BDCN-3h, these three broad peaks appear at the same positions. Due to the successful coordination of Co^2+^ and terephthalamide, Co-BDCN-24h could not dissolve in DMSO-d6. As a result, no resonance could be detected in the positions. Solid-state ^13^C NMR spectra of terephthalonitrile, terephthalamide, and Co-BDCN-24h are plotted. In contrast with the well-defined peaks of terephthalonitrile and terephthalamide (Fig. S2), the peaks of Co-BDCN-24h (Fig. 1c) are very broad (FWHM ≈ 200 ppm) due to the effect of paramagnetic Co^2+^ center.

**Fig. 2:**
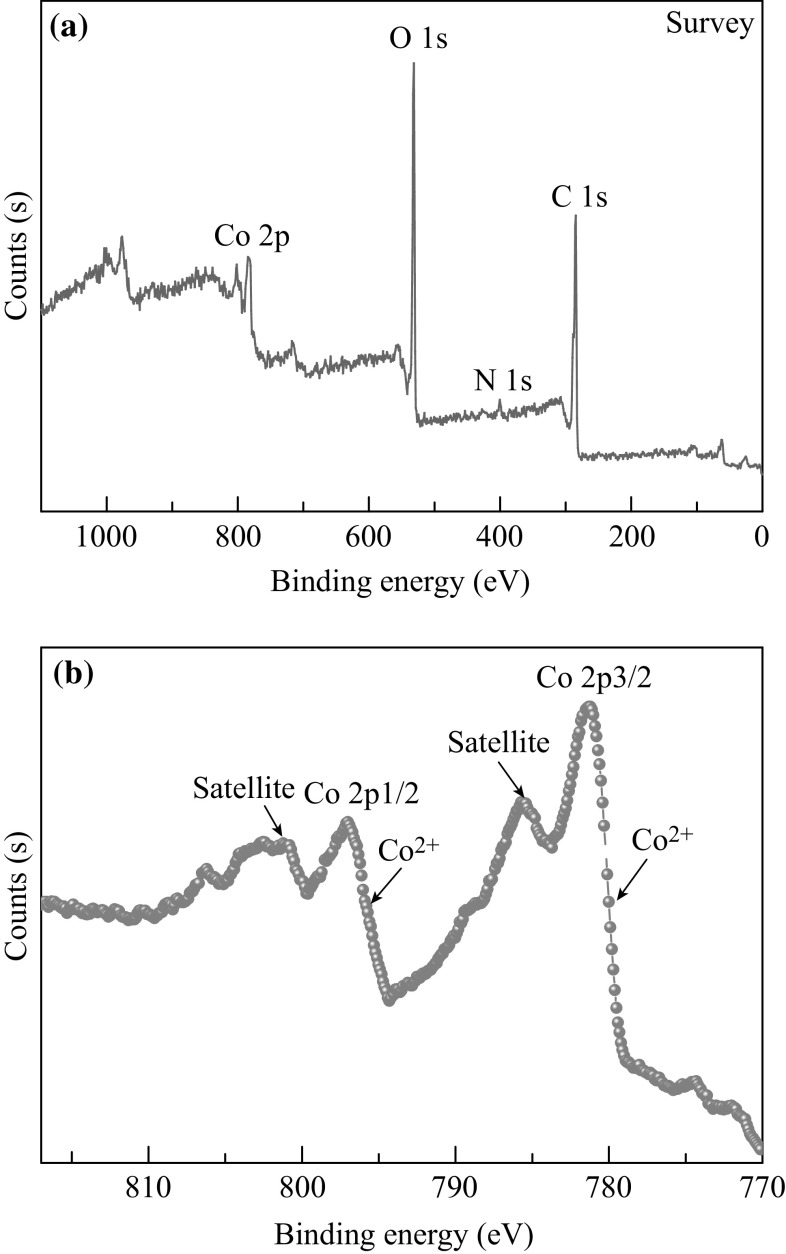
**a** XPS surveys and **b** high-resolution Co 2p XPS spectra of Co-BDCN-24h, from which the existence of C, N, O, and Co^2+^ was determined

TGA was used to investigate the thermal response of Co-BDCN-24h. As shown in Fig. 1d, before the decomposition, continuous weight loss corresponds to the loss of coordinated solvent or the free H_2_O molecules. Subsequently, rapid weight loss in TG curves demonstrates decomposition of the Co-BDCN-24h skeleton above 257 °C. After the decomposition of organic linkers is complete, at ∼ 314 °C, the residual material is converted to Co_3_O_4_. Finally, 46.5% of Co-BDCN-24h was retained, corresponding to 34.2% Co species. Furthermore, the form of Co^2+^ was also determined by XPS in Fig. 2.

**Fig. 3:**
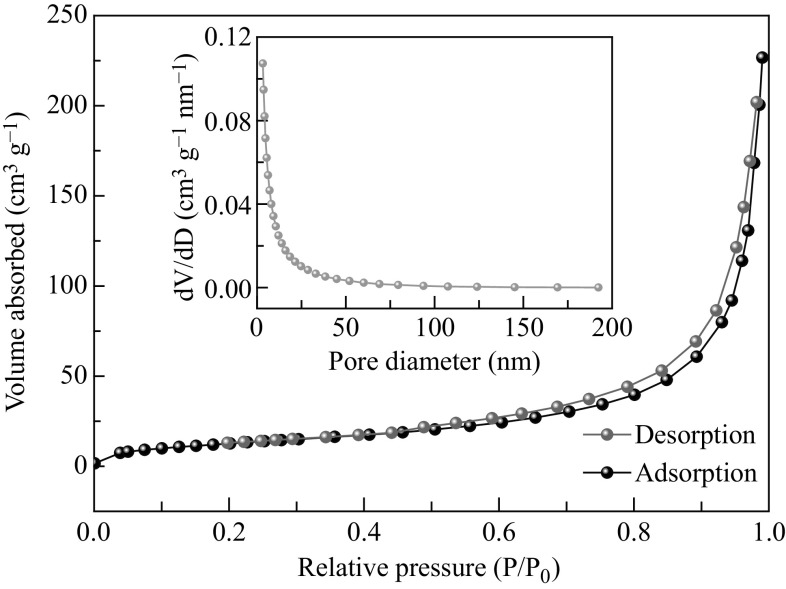
Nitrogen adsorption/desorption isotherm of Co-BDCN-24h. Inset: the pore-size distribution calculated based on the desorption branch of the corresponding isotherm

Nitrogen adsorption–desorption isotherms were measured at 77 K to determine the mean pore diameters and surface areas (Fig. 3). A mixed H3- and H1-type hysteresis loop of III isotherm reveals a combination of inter-particle and structural pores. Besides, a wide distribution of pore sizes (< 4–120 nm) is observed for Co-BDCN-24h, demonstrating the coexistence of mesopores and macropores. A surface area of 24.5 m^2^ g^−1^, a total pore volume of 0.35 cm^3^ g^−1^, and a mean pore diameter of 28.7 nm were determined by the Brunauer–Emmett–Teller (BET) method. Unlike traditional MOFs with ultra-high surface area, the moderate specific area of Co-BDCN-24h may weaken accessorial secondary reactions with the electrolyte [32, 42, 46].

**Fig. 4:**
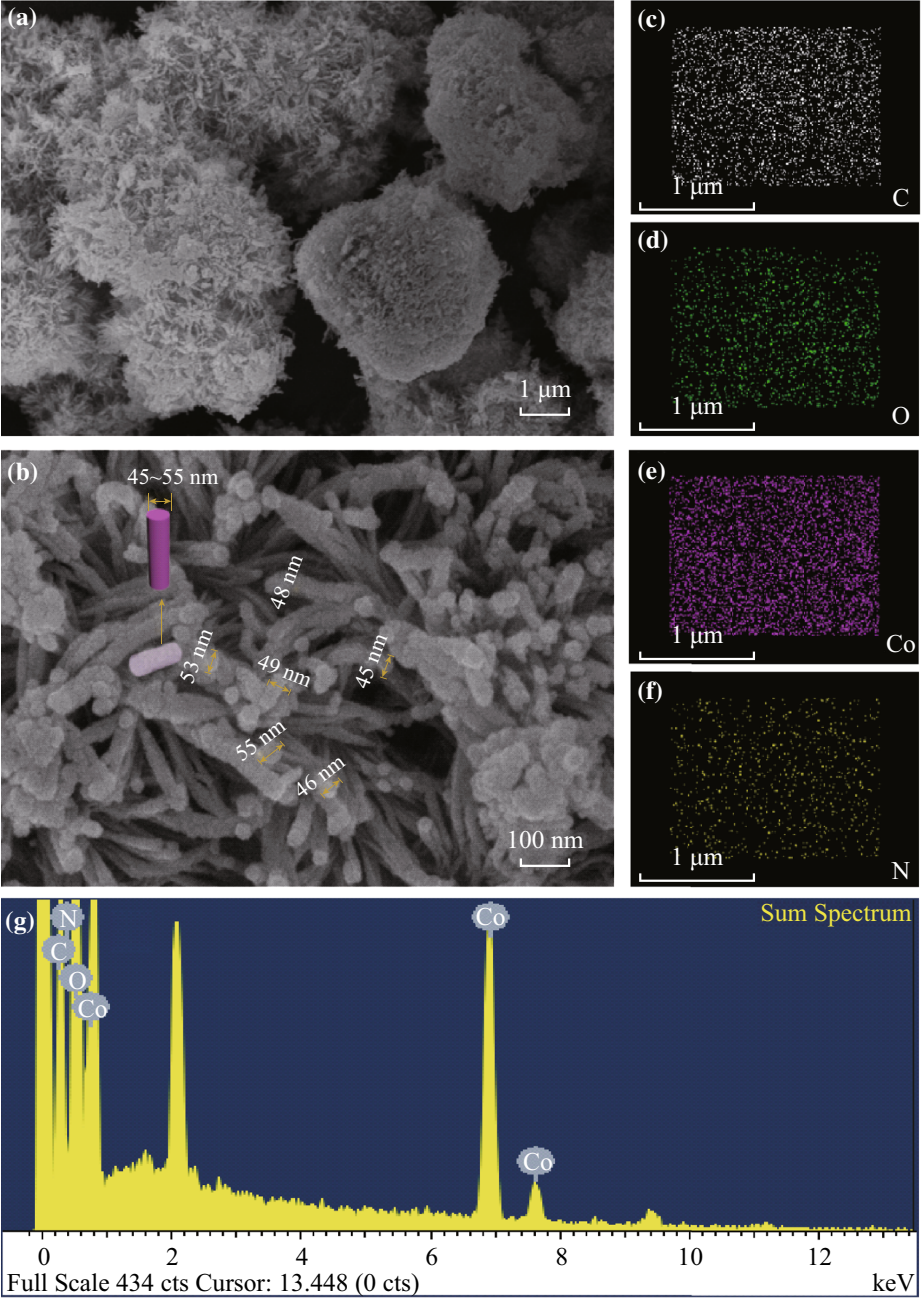
**a**, **b** SEM micrographs of the synthesized Co-BDCN-24h at low and high magnifications. EDS elemental mapping images (**c** C, **d** O, **e** Co, and **f** N) and **g** whole-energy spectra of Co-BDCN-24h. The peak at ~ 2 keV in the EDS spectrum is attributed to gold

Only three well-defined diffraction peaks of Co-BDCN-24, at 2*θ* = 10.1°, 11.2°, and 20.0°, were observed in PXRD patterns (Fig. S3), which indicates that most samples are present in the amorphous form. Figure 4a shows a full view of Co-BDCN-24h with uniform morphology, indicating a unified structure even in an amorphous state. Higher-magnification SEM images (Fig. 4b) reveal that Co-BDCN-24h is composed of nanowires with a diameter distribution of 45–55 nm. Elemental analysis using energy-dispersive X-ray spectroscopy (EDS) shows the homogeneous distribution of Co, O, C, and N in Co-BDCN-24h. On the basis of above-mentioned analysis, Co-BDCN-24h can be defined as an amorphous coordination polymer with nanowire morphology.

**Fig. 5:**
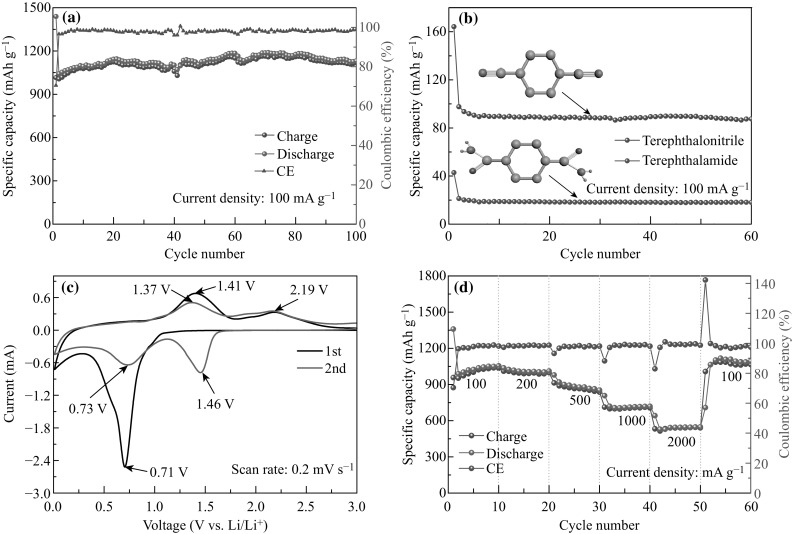
**a** Cycling performance and coulombic efficiency of Co-BDCN-24h at 100 mA g^−1^. **b** Cycling performance and coulombic efficiency of terephthalonitrile and terephthalamide at 100 mA g^−1^. **c** Cyclic voltammograms for the first two cycles of Co-BDCN-24h at a scan rate of 0.2 mV s^−1^. **d** Rate performance of Co-BDCN-24h

**Fig. 6:**
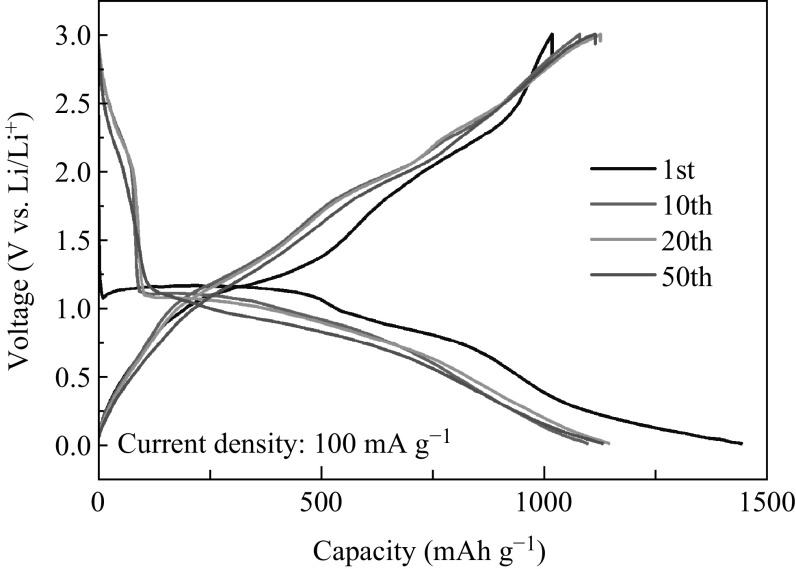
Galvanostatic charge–discharge profiles of Co-BDCN-24h at 100 mA g^−1^

The cycling performance and coulombic efficiency of Co-BDCN-24h were tested at 100 mA g^−1^ in a voltage range of 0.01–3.0 V versus Li/Li^+^. As shown in Fig. 5a, the initial cycle coulombic efficiency (CE) was calculated to be 70.54% by the discharge and charge capacities of 1439 and 1015 mAh g^−1^, respectively. As the side reactions of the electrode disappeared after several cycles, the subsequent charge/discharge curves are analogous (10th, 20th, and 50th, as shown in Fig. 6), indicating a reversible insertion/extraction of Li^+^. After 100 galvanostatic charge/discharge cycles, a reversible capacity of 1132 mAh g^−1^ was obtained. To the best of our knowledge, this should be one of the best LIB performances among MOF- and CP-based anode materials that operate at a rate of 100 mA g^−1^ (Table S1). Moreover, almost 100% of the coulombic efficiencies are retained in the subsequent cycles, indicating a facile intercalation/extraction of Li^+^ and an efficient transport of ions and electrons in Co-BDCN-24h. In contrast, when operated under the same test conditions, the reversible capacities of terephthalamide and terephthalonitrile are only 18 and 85 mAh g^−1^, respectively (Fig. 5b). Therefore, we suppose that the ultra-high stability of Co-BDCN-24h should be attributed to synergistic effects of organic linkers and metal centers.

**Scheme 2:**
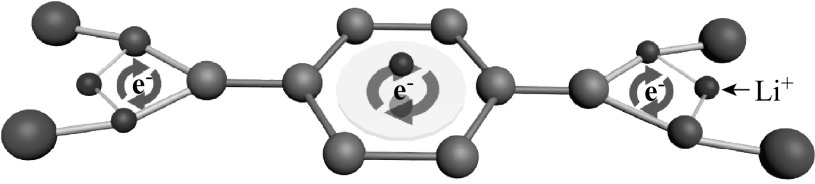
The schematic view of lithiation sites for Li^+^ adsorbed on benzene ring and amide group with the electron-donating effect of Co-BDCN-24h

The electrochemical behavior and the reaction mechanism of as-prepared Co-BDCN-24h were also studied by cyclic voltammetry (CV) measurements on 2032 cells in the voltage range of 0.01–3.0 V. Figure 5c presents the first two consecutive segments in the CV curves. A sharp cathodic peak is observed at ~ 0.71 V, which can be attributed to the associated electrolyte decomposition and the formation of SEI film on the surface of electrode. Two cathodic peaks at 0.73 and 1.46 V are observed in the subsequent sweep. The peaks were centered at 1.41–1.37 and 2.19 V during the anodic scans. The two reduction peaks in CV curves could be mainly attributed to insertion of Li^+^ to different organic moieties (benzene ring and amide group) [39, 42]. The electron-donating effect of the oxygen and nitrogen atoms in the amide groups, and that of the benzene ring, should be the main impetus in storing lithium ion (Scheme 2).

Rate performance was also studied to further explore the electrochemical capability of Co-BDCN-24h. Figure 5d shows the change of cycling performance with increasing rates: 100, 200, 500, 1000, and 2000 mA g^−1^. The charge capacities corresponding to these rates are 1000 ± 35, 1020 ± 30, 866 ± 13, 713 ± 7, and 538 ± 10 mAh g^−1^, respectively. After repeating the rate test at 100 mA g^−1^ for 50 cycles, the capacity is recovered with a value of about 1100 mA g^−1^ and is sustained at a steady value in the subsequent cycles, which indicates that the Co-BDCN-24h anode remains stable during the rate cycling process.

**Fig. 7:**
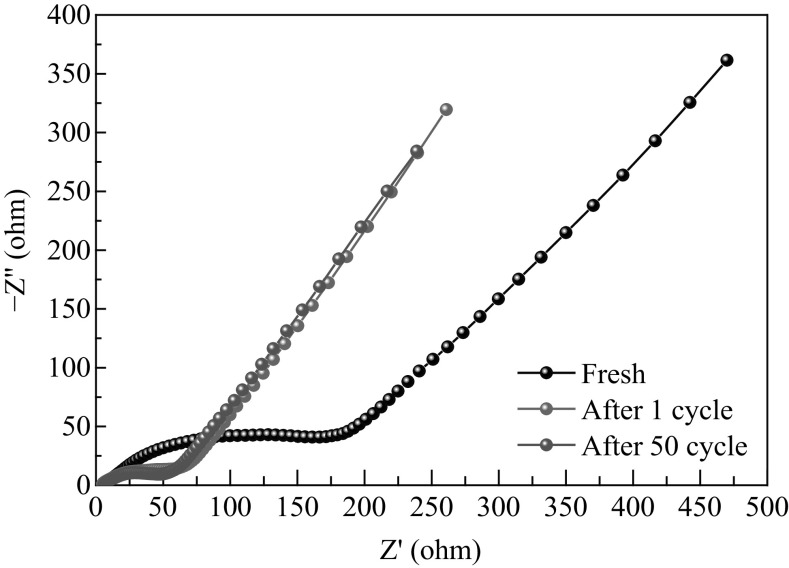
Nyquist plots of Co-BDCN electrode at different cycling intervals

**Fig. 8:**
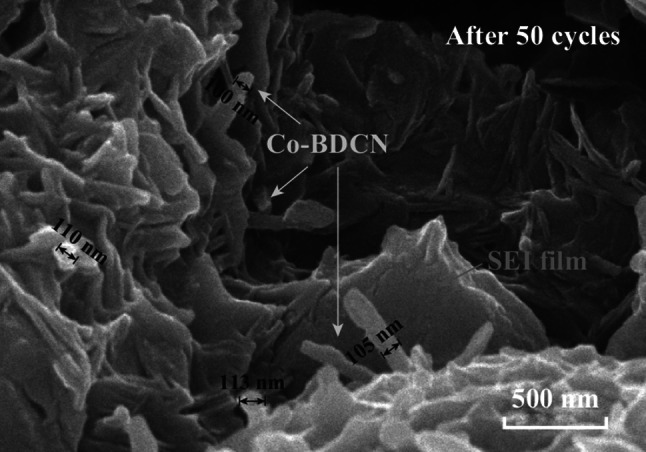
SEM micrograph of Co-BDCN electrode after 50 cycles

The Nyquist plots for a fresh sample of Co-BDCN-24h, measured after 1 and 50 cycles, are shown in Fig. 7. The frequency range was set between 0.01 Hz and 1 MHz with an AC amplitude of 10 mV. The solution resistances (*R*_s_) are 6.7, 4.6, and 5.9 Ω, respectively, while the charge transfer resistances (*R*_ct_) are 147.1, 57.0, and 50.4 Ω, respectively. The decrease in *R*_ct_ after the first cycle indicates an improved conductivity due to the activation and a better wetting of the electrodes. The small value of *R*_ct_ indicates good Li^+^ diffusion into the Co-BDCN-24h electrode. An ex situ SEM image of Co-BDCN-24h electrode at 0.01 V, which was taken after 50 cycles, is displayed in Fig. 8. Nanowire-like structures with a diameter of over 100 nm are observed, indicating that the initial morphology is preserved. The increase in diameter might be attributed to the Li^+^ intercalation into the Co-BDCN nanowire and the formation of SEI films (over 25 nm).

## Conclusion

In the past, CPs or MOFs based on carboxylate ligands, such as 1,3,5-benzenetricarboxylate and 1,4-benzenedicarboxylate, have shown potential for Li^+^ storage. In this work, an amide-group-based CP, Co-BDCN-24h, was synthesized and characterized for the first time. The Co-BDCN-24h electrode, with uniform nanowire morphology, demonstrated ultra-high capacity for Li^+^ storage, i.e., 1132 mAh g^−1^ at 100 mA g^−1^ (after 100 cycles). The great reversible capacity and superior cycling stability were attributed to the synergistic effect between metal centers and organic ligands, as well as the preservation of the nanowire morphology during cycling. This work provided an alternative to conjugated dicarboxylate-based MOF anode materials.

## Electronic supplementary material

Below is the link to the electronic supplementary material.

Supplementary material 1 (PDF 666 kb)
