# Supplementary material for: One-Pot Synthesis of Co-Based Coordination Polymer Nanowire for Li-Ion Batteries with Great Capacity and Stable Cycling Stability
Source: Nanomicro Lett. 2017 Dec 8;10(2):19. doi: 10.1007/s40820-017-0177-x (PMC6199072; doi:10.1007/s40820-017-0177-x)
Supplement: Supplementary file 1 — Supplementary material 1 (PDF 666 kb) [file 40820_2017_177_MOESM1_ESM.pdf]

Supporting Information for

One-pot Synthesis of Co-based Coordination Polymer Nanowire for Li-ion Batteries with Great Capacity and Stable Cycling Stability

Peng Wang[1], Xiaobing Lou[1], Chao Li[1], Xiaoshi Hu[1], Qi Yang[1,] *, Bingwen Hu[1]

[1]State Key Laboratory of Precision Spectroscopy, Shanghai Key Laboratory of Magnetic Resonance, School of Physics and Materials Science, East China Normal University, Shanghai 200062, People's Republic of China

∗Corresponding author. E-mail: qyang@lps.ecnu.edu.cn

Tel.: 86-21-62233171

Table and Figures

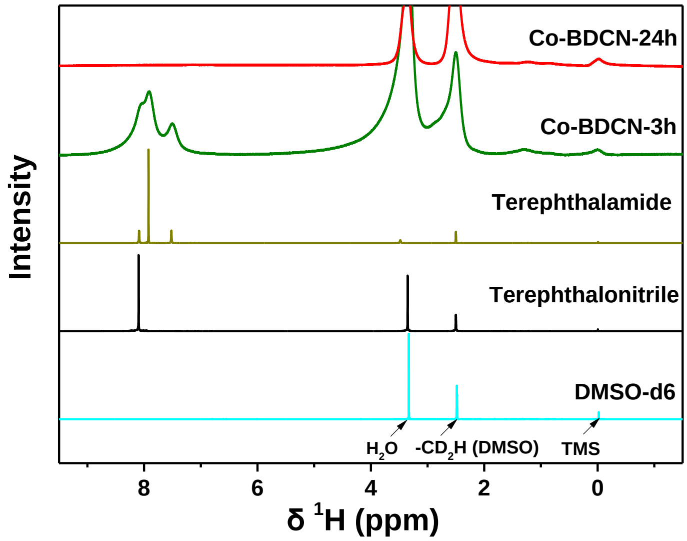

Fig. S1 ^{1}H NMR spectra of Co-BDCN-24h, Co-BDCN-3h, terephthalamide and terephthalonitrile in DMSO-d6 liquids

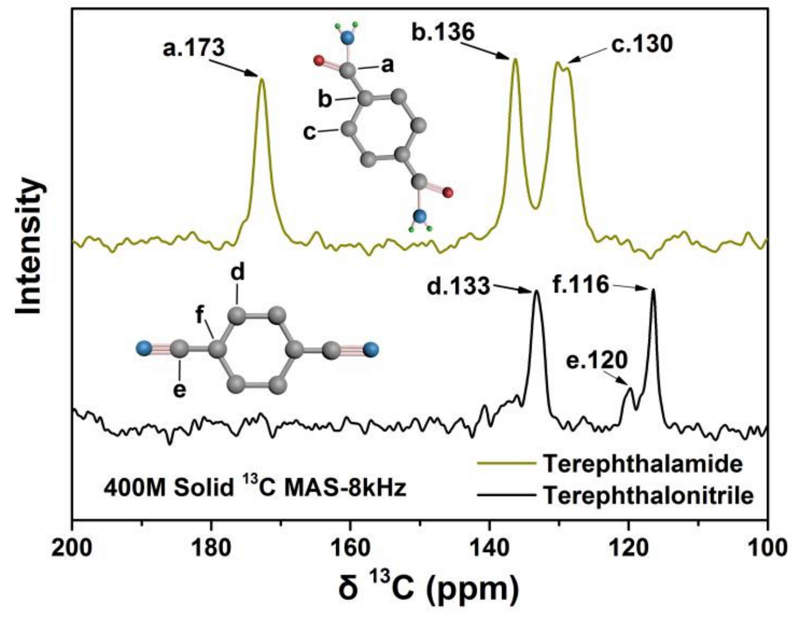

Fig. S2 ^{13}C NMR spectra of terephthalamide and terephthalonitrile in solid-state NMR

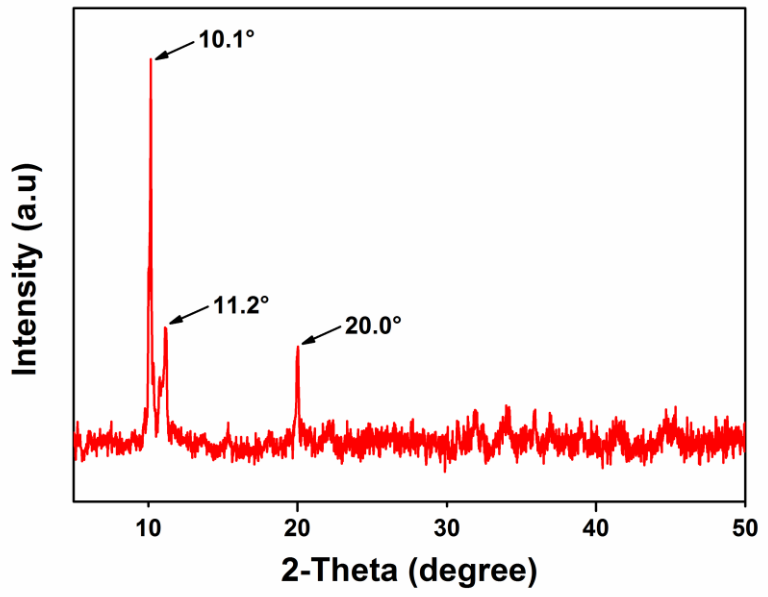

Fig. S3 XRD pattern of Co-BDCN-24h

Table S1 Metal organic frameworks (MOFs) or coordination polymers (CPs) for LIBs anode

organic ligands	MOFs or CPs	Voltage (V vs Li+/Li)	Current Density (mA g^{-1})	Cycle Number	Specific Capacity (mAh g^{-1})	Refs
H_2BDC	$Co_2(OH)_2BDC$	0.02–3.0	50	100	650	[1]
	Ni-MOF	0.01–3.0	100	100	620	[2]
	$[Cu_2(C_8H_4O_4)_4]_n$	0.01–2.5	48	50	161	[3]
H_3BTC	CoBTC-EtOH	0.01–3.0	100	100	856	[4]
	Mn-BTC	0.01–2.0	100	100	694	[5]
	$Cu_3(BTC)_2$	0.05–3.0	383	50	474	[6]
H_4BTC	MnCo-BTC	0.01–3.0	100	150	901	[7]
H_3BTB	MOF-177	0.1–1.6	50	2	425	[8]
IM & abIM	$Zn(IM)_{1.5}(abIM)_{0.5}$	0.01–3.0	100	200	190	[9]
H_2tfbdc & 4,4′-bpy	Mn-LCP	0.1–3.0	50	50	390	[10]
NTCDA	Ni-NTC	0.01–3.0	100	80	248	[11]
	Li-NTC	0.01–3.0	100	80	468	
	Li/Ni-NTC	0.01–3.0	100	80	482	
HCOOH	$Zn_3(HCOO)_6$	0.005–3.0	60	60	560	[12]
	$Co_3(HCOO)_6$	0.005–3.0	60	60	410	
	$Zn_{1.5}Co_{1.5}(HCOO)_6$	0.005–3.0	60	60	510	
4,4′-ocppy	[Pb(4,4′-ocppy)2]·7H2O	0.01-3.0	100	500	489	[13]
Terephthalamide	Co-BDCN	0.01–3.0	100	100	1132	This work

H_2BDC: 1,4-benzenedicarboxylate;
H_3BTC: 1,3,5-benzenetricarboxylate;
H_4BTC: 1,2,4,5-benzenetetracarboxylate;
H_3BTB: 1,3,5-benzenetribenzoate;
IM: Imidazole;
abIM: 2-aminobenzimidazole;
H_2tfbdc: 2,3,5,6-tetrafluoroterephthalic acid;
4,4-bpy: 4,4′-bipyridine;
NTCDA: 1,4,5,8-Naphthalenetetracarboxylic dianhydride;
HCOOH: methanoic acid.
4,4′-ocppy: 4-(4-carboxyphenyl)pyridine N-oxide

References

[1] L. Gou, L. Hao, Y.X. Shi, S. Ma, X. Fan, L. Xu, D. Li, K. Wang, One-pot synthesis of a metal-organic framework as an anode for Li-ion batteries with improved capacity and cycling stability. J. Solid State Chem. **210**(1), 121-124

(2014). https://doi.org/10.1016/j.jssc.2013.11.014
[2] Y. Zhang, Y. Niu, T. Liu, Y. Li, M. Wang, J. Hou, M. Xu, A nickel-based metal-organic framework: A novel optimized anode material for Li-ion batteries. Mater. Lett. **161**, 712-715 (2015). https://doi.org/10.1016/j.matlet.2015.09.079
[3] R. Senthil Kumar, C. Nithya, S. Gopukumar, M. Anbu Kulandainathan, Diamondoid-structured Cu-dicarboxylate-based metal-organic frameworks as high-capacity anodes for lithium-ion storage. Energy Technol. **2**(11), 921-927 (2014). https://doi.org/10.1002/ente.201402076
[4] C. Li, X. Lou, M. Shen, X. Hu, Z. Guo, Y. Wang, B. Hu, Q. Chen, High anodic performance of Co 1,3,5-benzenetricarboxylate coordination polymers for Li-ion battery. ACS Appl. Mater. Interfaces **8**(24), 15352-15360 (2016). https://doi.org/10.1021/acsami.6b03648
[5] S. Maiti, A. Pramanik, U. Manju, S. Mahanty, Reversible lithium storage in manganese 1,3,5-benzenetricarboxylate metal-organic framework with high capacity and rate performance. ACS Appl. Mater. Interfaces **7**(30), 16357-16363 (2015). https://doi.org/10.1021/acsami.5b03414
[6] S. Maiti, A. Pramanik, U. Manju, S. Mahanty, Cu_3 (1,3,5-benzenetricarboxylate)2 metal-organic framework: A promising anode material for lithium-ion battery. Microporous Mesoporous Mater. **226**, 353-359 (2016). https://doi.org/10.1016/j.micromeso.2016.02.011
[7] T. Li, C. Li, X. Hu, X. Lou, H. Hu, L. Pan, Q. Chen, M. Shen, B. Hu, Reversible lithium storage in manganese and cobalt 1,2,4,5-benzenetetracarboxylate metal-organic framework with high capacity. RSC Adv. **6**(66), 61319-61324 (2016). https://doi.org/10.1039/C6RA07727G
[8] C. Zhao, C. Shen, W. Han, Metal-organic nanofibers as anodes for lithium-ion batteries. RSC Adv. **5**(26), 20386-20389 (2015). https://doi.org/10.1039/C4RA16416D
[9] Y. Lin, Q. Zhang, C. Zhao, H. Li, C. Kong, C. Shen, L. Chen, An exceptionally stable functionalized metal-organic framework for lithium storage. Chem. Commun. **51**(4), 697-699 (2015). https://doi.org/10.1039/C4CC07149B
[10] Q. Liu, L. Yu, Y. Wang, Y. Ji, J. Horvat, M. Cheng, X. Jia, G. Wang, Manganese-based layered coordination polymer: synthesis, structural characterization, magnetic property, and electrochemical performance in lithium-ion batteries. Inorg. Chem. **52**(6), 2817-2822 (2013). https://doi.org/10.1021/ic301579g
[11] X. Han, F. Yi, T. Sun, J. Sun, Synthesis and electrochemical performance of Li and Ni 1,4,5,8-naphthalenetetracarboxylates as anodes for Li-ion batteries. Electrochem. Commun. **25**(1), 136-139 (2012). https://doi.org/10.1016/j.elecom.2012.09.014
[12] K. Saravanan, M. Nagarathinam, P. Balaya, J.J. Vittal, Lithium storage in a metal organic framework with diamondoid topology - a case study on metal formats. J. Mater. Chem. **20**(38), 8329-8335 (2010). https://doi.org/10.1039/c0jm01671c
[13] L. Hu, X. Lin, J. Mo, J. Lin, H. Gan, X. Yang, Y. Cai, Lead-based metal-organic framework with stable lithium anodic performance. Inorg. Chem. **56**(8), 4289-

4295 (2017). https://doi.org/10.1021/acs.inorgchem.6b02663
